# Supplementary material for: The Structural Characteristics of an Acidic Water-Soluble Polysaccharide from Bupleurum chinense DC and Its In Vivo Anti-Tumor Activity on H22 Tumor-Bearing Mice
Source: Polymers (Basel). 2022 Mar 11;14(6):1119. doi: 10.3390/polym14061119 (PMC8952506; doi:10.3390/polym14061119)
Supplement: Supplementary file 1 [file polymers-14-01119-s001.zip › polymers-1603371-supplementary.pdf]

Supplementary Materials

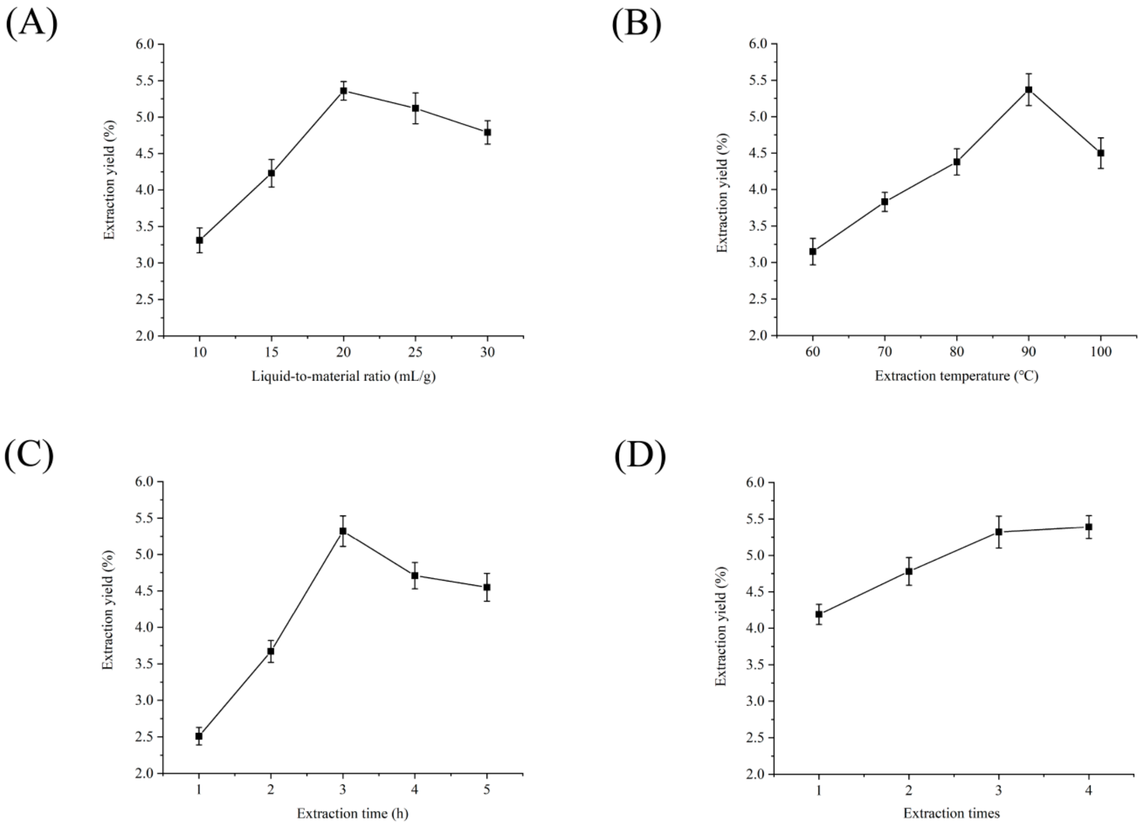

Figure S1. Effects of (A) liquid-to-material, (B) extraction temperature, (C) extraction time and (D) extraction times on the extraction yield of BCP (%).

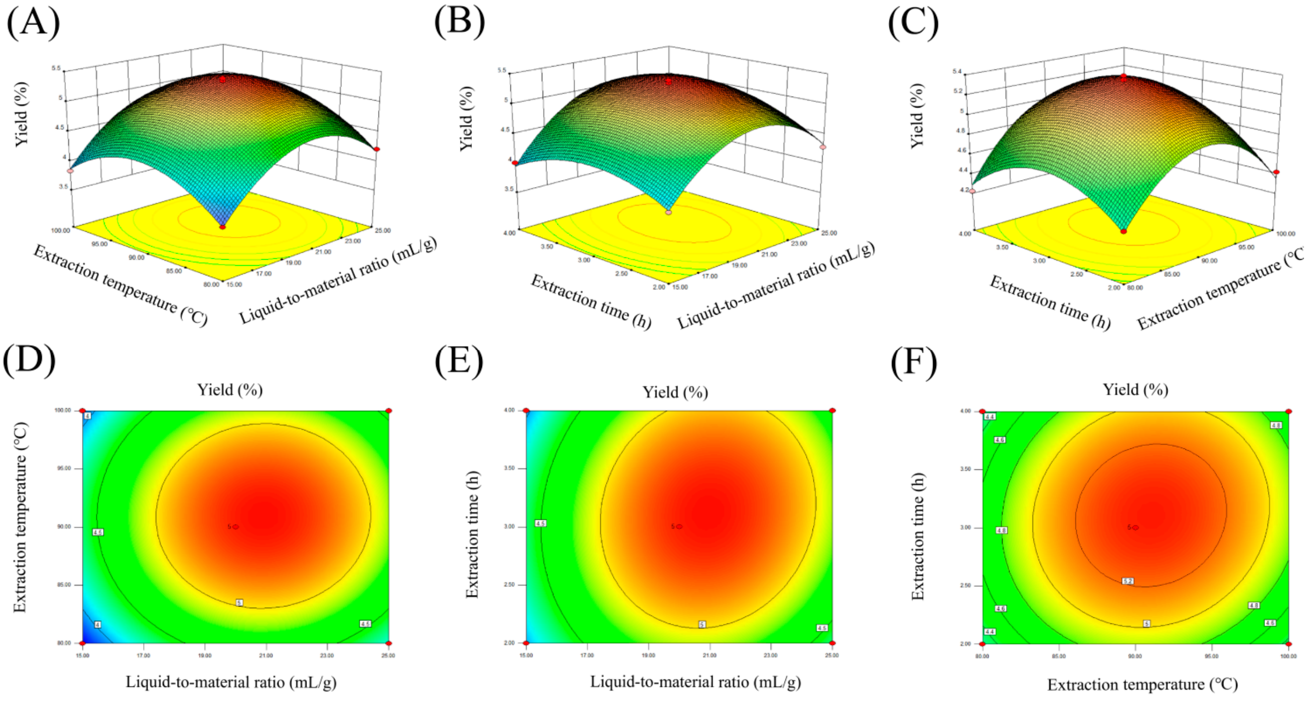

Figure S2. The 3-D response surface plots (A–C) and 2-D contour plots (D–F) showing the effects of ratio of liquid-to-material, extraction temperature and extraction time on the extraction yield of BCP and their mutual effects.

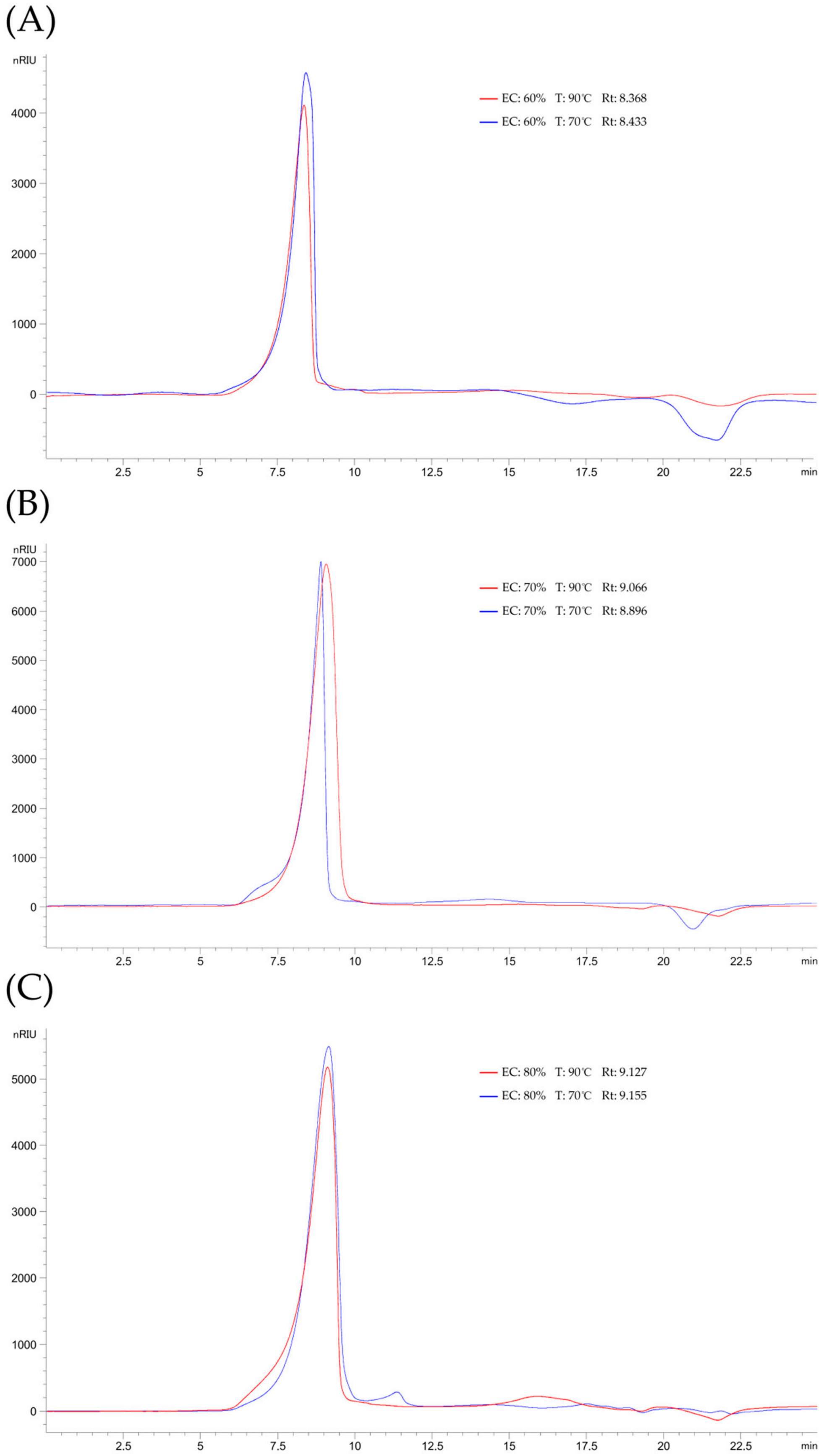

Figure S3. HPGPC chromatogram of six groups of *Bupleurum chinense DC* polysaccharides under differentethanol concentration (EC): 60% (A), 70% (B), 80% (C) and extraction temperature (T) 70 °C and 90 °C.

Table S1. Experimental design and results of Box-Behnken.

No.	A/ Liquid-to-Material ratio (mL/g)	B/ Extraction Temperature (◦C)	C/ Extraction Time (h)	Yield of BCP (%)
1	15	80	3	3.7
2	25	80	3	4.21
3	15	100	3	3.83
4	25	100	3	4.41
5	15	90	2	3.97
6	25	90	2	4.28
7	15	90	4	4.01
8	25	90	4	4.69
9	20	80	2	4.31
10	20	100	2	4.42
11	20	80	4	4.22
12	20	100	4	4.63
13	20	90	3	5.35
14	20	90	3	5.39
15	20	90	3	5.32
16	20	90	3	5.27
17	20	90	3	5.31

Table S2. ANOVA for the quadratic response surface model.

Source	Sum of squares	Degrees of freedom	Mean square	F-value	P-value	Significant level
Model	5.29	9	0.59	149.96	<0.0001	
A	0.54	1	0.54	137.88	<0.0001	**
B	0.090	1	0.090	23.03	0.0020	**
C	0.041	1	0.041	10.35	0.0147	*
AB	1.225E-003	1	1.225E-003	0.31	0.5937	
AC	0.034	1	0.034	8.73	0.0213	*
BC	0.022	1	0.022	5.74	0.0478	*
A2	2.21	1	2.21	562.72	<0.0001	**
B2	1.35	1	1.35	344.52	<0.0001	**
C2	0.57	1	0.57	144.20	<0.0001	**
Residual	0.027	7	3.922E-003	-		
Lack of Fit	0.019	3	6.458E-003	3.20	0.1455	
Pure error	8.080E-003	4	2.020E-003			
Cor Total	5.32	16				

A: Liquid-to-Material ratio(mL/g), B: extraction temperature (◦C), C: extraction time (h).

ANOVA: One-way analysis of variance.

$Y = 5.33+0.26A+0.11B+0.071C+0.017AB+0.093AC+0.075BC-0.72A^2-0.57B^2-0.37C^2$

R^2 =0.9948, Adj-R^2 = 0.9882

* Indicates significant differences ($P < 0.05$).

** Indicates extremely significant differences ($P < 0.01$)

Table S3. The monosaccharide compositions of *Bupleurum* polysaccharide

Name	Monosaccharide Composition (mol%)	Reference
BCP	Rha: Ara: Gal: Glu: GalA = 0.063:0.788:0.841:1:0.196	-
BCPS-1	Ara; Gal; Glc = 2.1:2.5:1	12
BC-PSI	Gal: GalA: Glc: Ara: Man =1.6:1.1:1.8:1.7:1.0	42
BCAP-1	Ara: Xyl: Man: Glc: GlcA =1.0:3.2:0.7:3.6:1.2	15
BCP	Ara: Xyl: Man: Glc =2.1:1:1.7:3.2	16
BC-PS2	Rha: Ara: Gal: Man = 3.5 : 2.4 : 2.0: 1.0	43
WBCP-A2	GalA: Gal: Ara: Rha: GlcA = 56.7:9.8:20.0:7.0:1.4	44
VBCP-3-A	Rha: Ara: Gal = 1.72:2.21:1.00	45
BCP	Man: Rha: GlcA: GalA: Glc: Gal: Xyl: Ara= 2.93: 2.62: 1.00: 4.57: 15.11: 23.28: 1.46: 25.34	11
BCPS-m	Ara: Gal: Glu= 5.3:0.6:1	46
